# Case study of 3D fingerprints applications

**DOI:** 10.1371/journal.pone.0175261

**Published:** 2017-04-11

**Authors:** Feng Liu, Jinrong Liang, Linlin Shen, Meng Yang, David Zhang, Zhihui Lai

**Affiliations:** 1 Computer Vision Institute, School of Computer Science & Software Engineering, Shenzhen University, Shen Zhen, Guang Dong, China; 2 Department of Computing, The Hong Kong Polytechnic University, Hung Hom, Kowloon, Hong Kong; University of Texas at San Antonio, UNITED STATES

## Abstract

Human fingers are 3D objects. More information will be provided if three dimensional (3D) fingerprints are available compared with two dimensional (2D) fingerprints. Thus, this paper firstly collected 3D finger point cloud data by Structured-light Illumination method. Additional features from 3D fingerprint images are then studied and extracted. The applications of these features are finally discussed. A series of experiments are conducted to demonstrate the helpfulness of 3D information to fingerprint recognition. Results show that a quick alignment can be easily implemented under the guidance of 3D finger shape feature even though this feature does not work for fingerprint recognition directly. The newly defined distinctive 3D shape ridge feature can be used for personal authentication with Equal Error Rate (EER) of ~8.3%. Also, it is helpful to remove false core point. Furthermore, a promising of EER ~1.3% is realized by combining this feature with 2D features for fingerprint recognition which indicates the prospect of 3D fingerprint recognition.

## Introduction

As one of the most widely used biometrics, fingerprints have been investigated for more than a century [1]. Meanwhile, with the rapid development of fingerprint acquisition devices and the advent of advanced fingerprint recognition algorithms, effective Automated Fingerprint Recognition Systems (AFRSs) are available in the market. However, they are almost based on 2D fingerprint features, even though the fact is that human fingers are 3D objects. Distortions and deformations will be introduced, while 3D information will be loss when 2D fingerprint images are used, which degrades the performance of AFRSs.

3D fingerprints come into researchers’ view in recent years with the expansion of acquisition technology [2–14]. Most of the work focused on the acquisition and preprocessing of 3D fingerprints, or at the most expanded two dimensional fingerprint features into 3D space but no recognition results were given [2–10]. Until 2015, researchers from Biometric Research Centre, the Hong Kong Polytechnic University [13, 14] began to investigate the utility of 3D fingerprint features and reported some experimental results of user authentication using the acquired biometric information. In [13], new low level of fingerprint features were proposed and used as additional features to fingerprint recognition. In [14], the authors reported the result of 3D minutiae matching, as well as the matching result of 3D curvature features. In both of those studies, low Equal Error Rate (EER) (from 13% to 35%) was obtained when features from 3D fingerprint images were used for recognition. The EER was not high either for 3D minutiae matching (around 10% when unknown users were added). There may be two reasons why those features are not so effective. One is that both of the mentioned 3D fingerprint images were reconstructed by 3D reconstruction techniques. The accuracy of 3D finger shape is degraded by reconstruction algorithms. The other one is that the distinctiveness of features extracted from 3D finger shape is lower than traditional three levels of features on 2D fingerprint images. However, the EER was greatly improved if those features were combined with 2D traditional features extracted from 2D fingerprint images for matching. Thus, there is no doubt that higher accuracy will be achieved if 3D fingerprint images are used for recognition.

This paper is motivated by analyzing the advantages and disadvantages of current 3D fingerprint recognition techniques. The contributions of this paper include two parts: (i). More accurate 3D finger shape was obtained by using Structured-light Illumination (SLI) method. The SLI method is widely used as a 3D imaging method for its high accuracy, speed and stability. (ii). Features on 3D fingerprint images were studied thoroughly and detailedly. By analyzing their distinctiveness, 3D fingerprint features can be used for different applications. It can be found that: (1) an EER of ~15% can be achieved if the whole 3D finger point cloud data are taken as 3D shape features for fingerprint recognition. However, it is very effective to align two fingerprints in a short time. (2) The proposed distinctive 3D shape ridge feature are suitable for assisting fingerprint recognition and also helpful to remove false core point. Fusion strategy is employed to combine 2D and 3D fingerprint matching results to figure out the effectiveness of improving recognition accuracy by including 3D fingerprint features. An EER of ~8.3% can be obtained when only distinctive 3D shape ridge feature is used for fingerprint recognition, while EER of ~1.3% is achieved by combing this 3D feature with 2D fingerprint features.

The organization of this paper is as follows. In Section 2, the acquisition and preprocessing of 3D finger point cloud data is introduced. 3D fingerprint features are investigated in Section 3. The corresponding feature extraction and matching algorithms are also given in Section 3. The applications and experimental results are shown in Section 4. Section 5 finally concludes this work and suggests the future research.

## 3D finger point cloud data acquisition and preprocessing

Currently, there are mainly two ways to generate 3D fingerprints. One is 3D reconstruction technique and the other one is Structured-light scanning [6, 15, 16]. Generally speaking, the 3D reconstruction technique has the advantage of low cost. However, it has the disadvantage of low accuracy, because it is difficult to find and match corresponding point pairs in two or more images [14, 17]. Structured-light imaging has high accuracy as well as a moderate cost [6, 15, 16]. Considering the requirements of accuracy for biometric authentication, we choose to use structured-light scanning to acquire the finger depth information.

**Fig 1:**
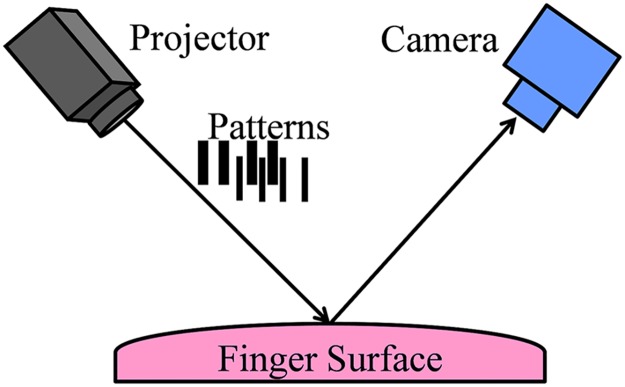
Schematic diagram of device used to capture 3D point cloud data of human fingers.

**Fig 2:**
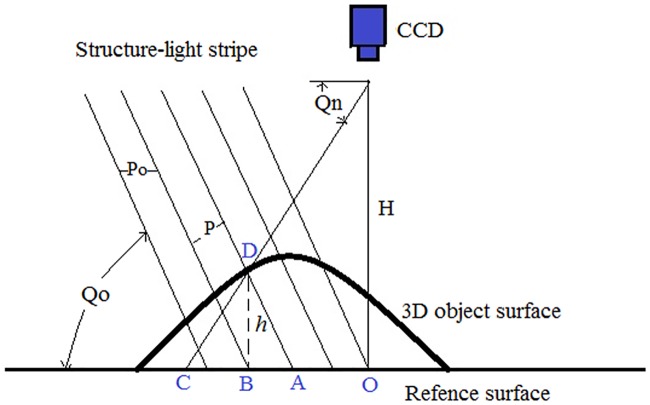
The principle of structured-light imaging.

As we all know, structured-light imaging is widely used in 3D imaging for its high accuracy, speed and stability. The structure diagram of the collection device is shown in Fig 1. A projector casts 13 structured-light stripes onto finger surface, the structured-light is modulated by the surface shape, and the modulated stripes are captured by a CCD camera at a constant distance from the projector. The distance from the measured surface to the reference plane can be calculated according to the modulated stripe images and the geometric correlation between the measured surface, projector and CCD camera. The structure diagram of the collection device is shown in Fig 1. Fig 2 illustrates the principle of the adopted structure light imaging technique. In Fig 2, the relative height of point D at spatial position (*x*, *y*) on the 3D object surface can be calculated by following Eq (1) [16]. Where the height of the reference surface is defined as 0. *P*_*0*_ is the wavelength of the projected light on the reference surface, *Q*_*0*_ is the projecting angle, *Q*_*n*_ is the angle between the reference surface and the line which passes through the current point and the CCD center, and *ϕ*_*CD*_ is the phase difference between points *C* and *D*. Since the phase of point *D* is equal to the phase of point *A* on the reference surface, *ϕ*_*CD*_ can be calculated as Eq (2).

$$h(x,y)=\bar{BD}=\frac{P_{0}\cdot \text{tan}Q_{0}\cdot \phi_{CD}}{2\pi (1+\text{tan}Q_{0}/\text{tan}Q_{n})}$$ (1)

$$\phi_{CD}=\phi_{CA}=\phi_{OC}-\phi_{OA}$$ (2)

**Fig 3:**
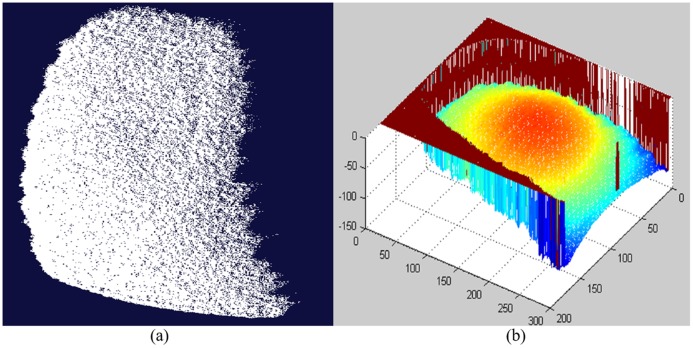
An example of 3D point cloud data of a human finger shown by (a) 3D display software, (b) MATLAB display tool.

The depth information of the 3D object surface can be retrieved by using Eq (1) and the phase shifting and unwrapping technique in [16]. Interested readers can refer to [15, 16] for more details. With this processing, the relative height of each point *h(x*, *y)* could be calculated. The range data of the finger surface can then be obtained. Fig 3 shows an example of finger point cloud data we obtained. In the captured data, the size of the 3D image is 640×480 with about 380 dpi resolution, i.e., there are totally 307,200 cloud points to represent the 3D fingerprint information.

**Fig 4:**
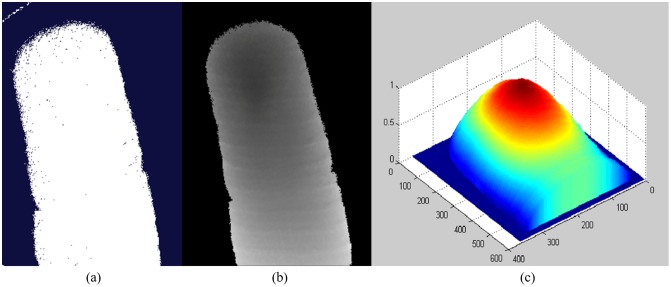
Preprocessing procedure. (a) Original 3D finger point cloud data, (b) Extracted ROI in 2D plane, (c) Pre-processed 3D finger point cloud data.

Since it is inevitable to introduce noises (e.g. false estimated depth near the edge of Region of Interest (ROI), missing or misestimated depth in ROI) when data was collected, it is necessary to pre-process the original point cloud data. In this paper, the point cloud data was firstly projected into 2D plane, i.e. a 3D point (*x*, *y*, *z*) corresponds to a point (*row*, *column and intensity*) on a figure, as shown in Fig 4(a). Then, the morphological operators (e.g. interpolation of inner missed points, edge corrosion) provided by MATLAB toolbox were adopted to remove edge points and fill in the inner points. As shown in Fig 4(b), inner missed points and edge points are removed when it was compared with Fig 4(a). The area with the largest size in the figure was chosen as the ROI of the finger. Then, Gaussian smoothing algorithm with window size of 5 × 5 pixels was used to process the missed or misestimated depth points in ROI. Simple MAX-MIN rule was finally employed to normalize the depth value into [0, 1], the preprocessed 3D point cloud data of fingerprint is shown in Fig 4(c). The normalized depth value (*W*.*norm*) can be calculated: *W*.*norm* = (*W*–min (*W*)) / (max (*W*) − min (*W*)) when the MAX-MIN rule [18] is used. Where *W* is the set of depth value.

## Investigation of 3D fingerprint features

**Fig 5:**
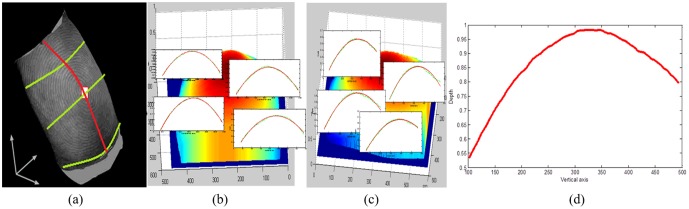
A fingerprint image in 3D space. (a) Fingerprint image with texture in 3D space, (b) 3D finger point cloud data and its horizontal sectional profiles, (c) 3D finger point cloud data and its vertical sectional profiles, (d) Distinctive 3D shape ridge feature defined in the paper.

After preprocessing, stable and unique features are expected to be extracted for further application. Through observation (Fig 5), we found that 3D depth information reflects the overall structure of human finger. However, there are many invalid points in the whole 3D finger shape due to the structure of human finger. As shown in Fig 5(b), the horizontal sectional profiles are almost parabola-like curves in different fractions (the real data are labelled in green dashed line and the fitted data are labelled in red solid line in Fig 5(b)). The vertical profiles are also fitted by parabolic equation (red labelled in Fig 5(c)), it can be seen that most of lines can be well fitted by parabola, however, the closer the line near the highest point, the less like the real line to parabola. Thus, we extracted this line in the paper, and defined it as the distinctive 3D shape ridge feature. The detailed method to extract the distinctive 3D shape ridge feature is as follows: i). Fit each horizontal sectional profile by a parabolic equation; ii). Find the maximum value of each fitted line; iii). Connect all of maximum values to form a vertical line, namely the distinctive 3D shape ridge feature. Fig 5(d) shows the distinctive 3D shape ridge feature extracted using the proposed method. The method to extract the distinctive 3D shape ridge feature can be formulated as Eq (3). Where *x* is the variable of row-coordinate of the image, and *y* is the variable of column-coordinate of the image. *N*_*row*_ × *M*_*column*_ is the size of the image. *a*_*x*_, *b*_*x*_, and *c*_*x*_ represent the coefficients of the fitting parabola functions.

$$\begin{array}{l} F=\{\begin{array}{ccc} f_{x,y}, & x=1,2,\cdots N_{row}, & y=1,2,\cdots M_{column} \end{array}\}\\ f_{x,y}=f\left(x,\frac{4a_{x}c_{x}-b_{x}{}^{2}}{4a_{x}}\right)\\ f\left(x,y\right)=\begin{array}{cc} a_{x}y^{2}+b_{x}y+c_{x}, & y=1,2,\cdots M_{column} \end{array} \end{array}$$ (3)

**Fig 6:**
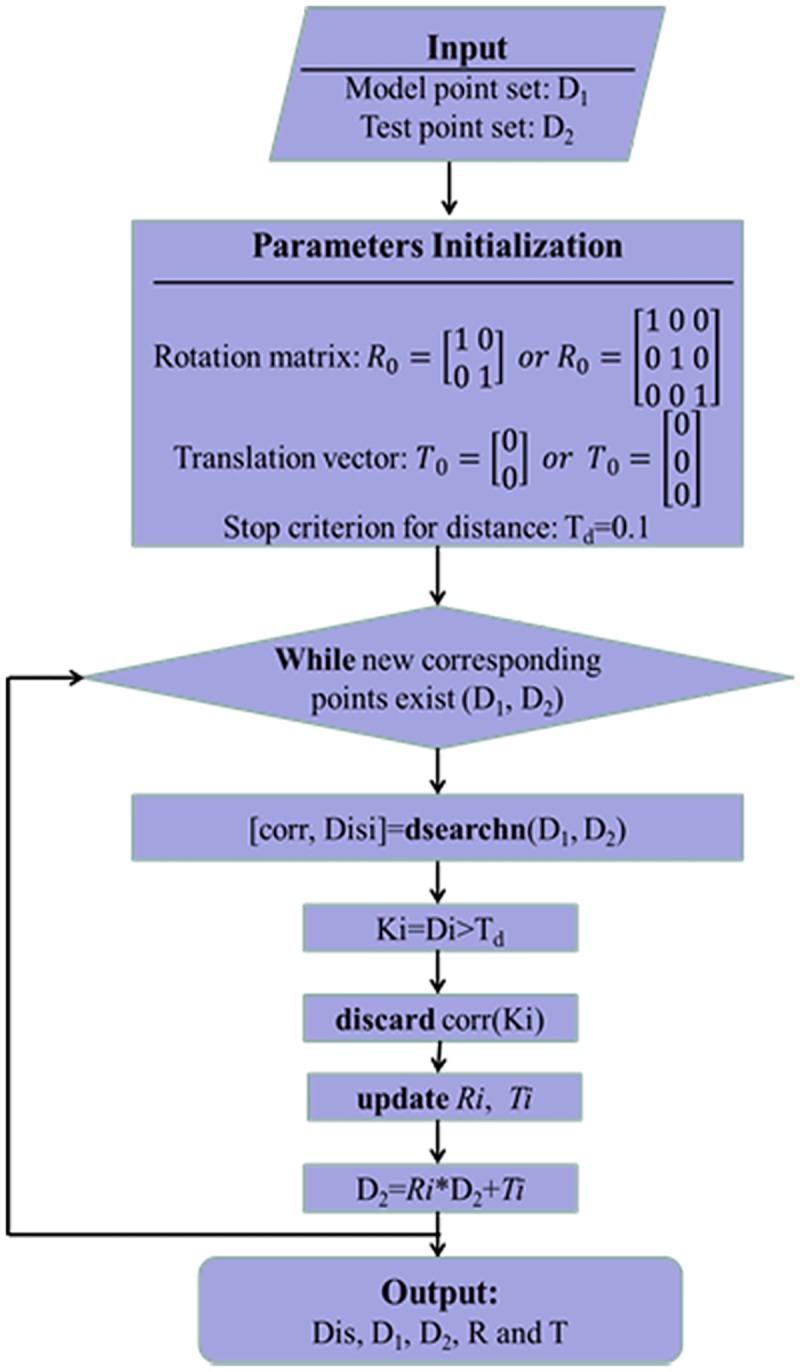
The flowchart of the ICP algorithm.

In addition, we introduced the matching method used in this paper here since it is very simple and classical. Intuitively, the iterative closest point (ICP) algorithm is suitable for solving such matching problem. ICP method [19] is widely used in many 2D image and 3D object recognition systems for matching. In this paper, we slightly modified the ICP method to measure the distances between two sets of points. The flowchart of the algorithm is given in Fig 6.

## Applications and experimental results

### 3D fingerprint applications and the experimental database

As described in detail previously [20, 21], a minutia feature in a 2D fingerprint image can be extended to 3D space. (e.g. in 2D: location and orientation, in 3D:, where x, y and z is the spatial coordinates. and is the orientation of the ridge). Thus, fingerprint recognition with higher security can be achieved by matching features in 3D space (e.g. 3D minutia matching [14, 22]).

This paper tried to discover the additional 3D fingerprint features and their applications to fingerprint recognition. Experiments were implemented in our own established 3D fingerprint database. The database contains 440 samples from 22 volunteers, including 12 males and 10 females between 20 to 40 years old. The 3D fingerprint samples were collected in two separate sessions, and in each session, each finger of a person was taken as one subject and was collected once. i.e. the database contains 440 samples from 220 fingers, 2 pictures for each finger (part of the database can be downloaded from S1 Database.zip). The average time interval between the two sessions was about two weeks. It is noted that the data were collected under the guidance of the collector to ensure the frontal view of each finger was taken. Three cases were summarized and studied in the paper, as shown in the following subsections.

### Case 1: Coarse alignment by 3D finger point cloud data

**Fig 7:**
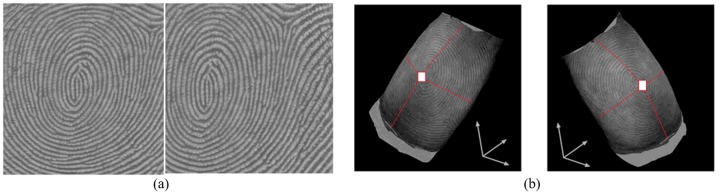
Comparison of 2D fingerprint image and 3D fingerprint image in fingerprint coarse alignment. (a) Two 2D images of the same finger, (b) Two 3D images of the same finger. er. (cited from our earlier work in [20]).

Coarse alignment is a crucial step in fingerprint recognition, especially for fingerprint matching. In 2D fingerprint images (Fig 7(a)), finger skin is forced to plane. Level-1 fingerprint features (e.g. orientation map, core point) are usually extracted at first for coarse alignment in fingerprint recognition [23–27].

Obviously, it takes some time to extract fingerprint features, and the accuracy is also affected by the results of feature extraction. While in 3D fingerprint images, as shown in Fig 7(b), two images can be aligned quickly by aligning their corresponding finger shapes. This is because the center part of the human finger is higher than the side part in 3D space from the frontal view of the finger. By making full use of this character, 3D fingerprint images can be quickly coarse aligned before matching in fingerprint recognition.

**Fig 8:**
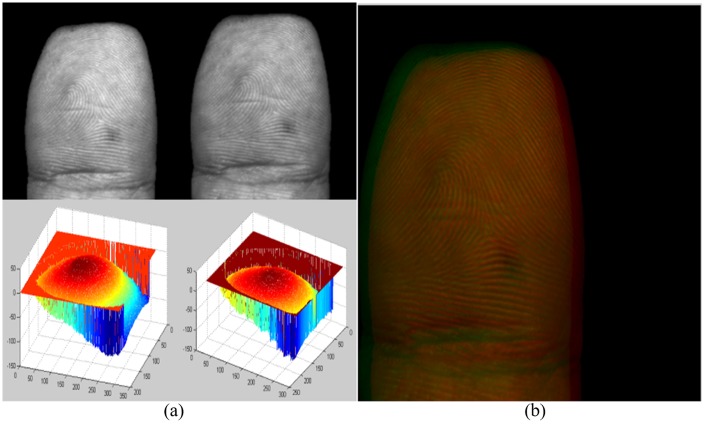
An example of coarse alignment results in our database. (a) Texture and 3D finger point cloud data of a template image and a test image, (b) Coarse alignment result of the template image and the test image. ((b) is cited from our earlier work in [20]).

**Fig 9:**
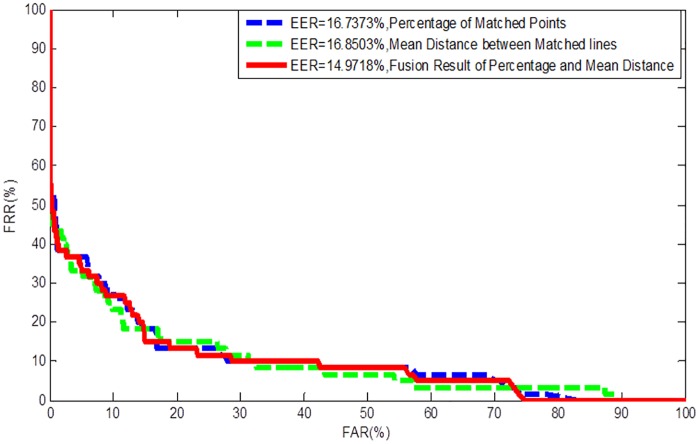
ROCs by matching the 3D finger point cloud data under different match score indexes.

Fig 8 shows an example of coarse alignment result according to their corresponding 3D finger point cloud data in our database. We also tried to match this feature directly to see its discriminative performance. This feature was matched by 3D ICP method introduced in Section 3 since it is 3D point cloud data. The mean distance between matched pairs (M*dist*) and the percentage of matched points (P*m* = matched pair number/overall pairs to be matched) are taken as the match score. The EERs were obtained from 220 genuine scores and 48,180 imposter scores (generated from 220 fingers, 2 pictures of each finger). Fig 9 shows the Receiver Operating Characteristic Curves (ROCs) of different match score indexes evaluated on the established database. It can be seen from the result that the EERs are all very high (best one: ~15%), which shows the identifiability of this feature is not very high. However, it is no doubt that this feature can be used for coarse alignment.

### Case 2: Core point detection under the guidance of distinctive 3D shape ridge feature

**Fig 10:**
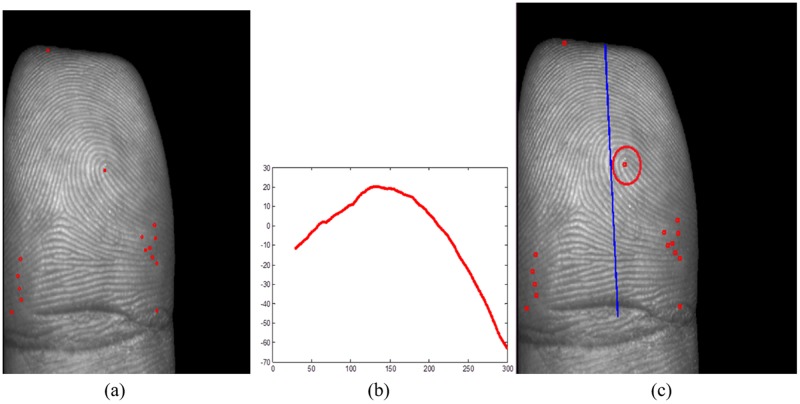
An example of core point detection. (a) Original core point set detected Poincare Index (red point), (b) The extracted distinctive 3D shape ridge feature, (c) Location of true core point under the guidance of distinctive 3D shape ridge feature (red circled). ((a) and (c) are cited from our earlier work in [20]).

**Fig 11:**
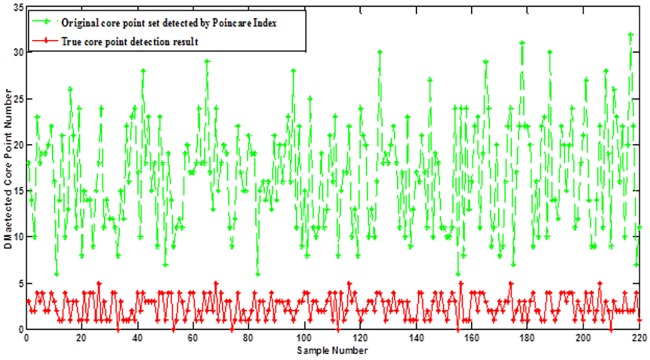
The statistical result of true core point detection from original core point set detected by Poincare Index in the established database with 220 samples.

It is interesting to find that core points in fingerprint images almost locate at the center part of the finger with highest curvature by observing human fingers. Thus, the position of the core point will be more likely located near the distinctive 3D shape ridge feature we defined in this paper. By making full use of the distinctive 3D shape ridge feature, false core points which are far away from the distinctive 3D shape ridge feature can be easily removed. Fig 10(a) shows an example of core point detection results by Poincare Index introduced in [1]. It can be seen that many false core points are detected by using this method. We then used the extracted distinctive 3D shape ridge feature (Fig 10(b)) to guide the location of the true core point. The final result is given in Fig 10(c), which shows the helpfulness of the usage of distinctive 3D shape ridge feature to true core point detection. The statistical result of true core point detection from original core point set detected by Poincare Index in our established database with 220 samples (each finger selects one picture) is shown in Fig 11. From the result, it can be seen that most of the false core points are removed from original core point set even though not only the true core point is detected each time, which fully demonstrates the effectiveness of using the distinctive 3D shape ridge feature to true core point detection.

### Case 3: User authentication using distinctive 3D shape ridge feature

**Fig 12:**
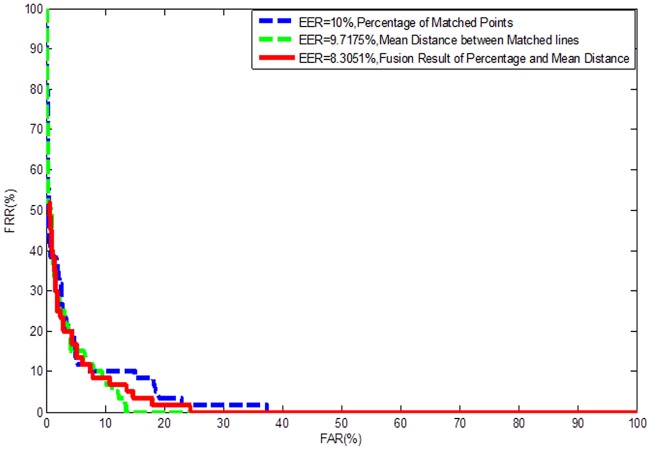
ROCs by matching the distinctive 3D shape ridge feature under different match score indexes.

As mentioned in Section 3, the defined distinctive 3D shape ridge feature is in fact a representative vertical section profile of 3D finger point cloud data. The dimension of this feature is reduced from 3 to 2. Thus, this feature can be matched by 2D ICP. In the study, M*dist* and P*m* mentioned in subsection 4.2 are taken as the match score, too. Fig 12 shows the ROCs of different match score indexes evaluated on the established database. From the results, we can see that the index of mean distance between matched pairs is better than the percentage of matched points. Best EER of around 8.3% can be obtained when the distinctive 3D shape ridge feature was used for matching by simple ICP algorithm, which demonstrates this feature is helpful to distinguish different fingers even though it is not as accurate as other higher level fingerprint features.

Since both 2D fingerprint images and 3D finger point cloud data of the same finger are provided simultaneously by 3D fingerprints, we tried to study whether improved performance can be achieved by combining 2D and 3D fingerprint features or not. Here, for 2D fingerprint features, the Distal Interphalangeal Crease based (DIP-based) feature was selected due to its effectiveness on our database compared with minutiae and Scale Invariant Feature Transformation (SIFT) features [28]. The angular distance was used as the match scores (*MS*_*2D*_). DIP is defined as the only permanent flexion crease which is located between medial and distal segments of finger except thumb (between proximal and distal segments), and the DIP-based feature was formed by coding the region using competitive coding scheme introduced in [29]. The detailed DIP feature extraction and matching algorithms can be obtained in [28] which was attached as supporting information. Meanwhile, the mean distance between matched pairs be matching the defined distinctive 3D shape ridge feature was taken as the match score (*MS*_*3D*_). A simple adaptive weighted sum rule is used to combine the 2D and 3D matching scores. The combined score can be expressed as:

$$\begin{array}{cccc} MS_{2D+3D} & = & w/MS_{2D}+\left(1-w\right)/MS_{3D} & w\in \left[0,1\right] \end{array}$$ (4)

The weight w is adaptively tuned to provide the best verification results at step length of 0.01.

**Fig 13:**
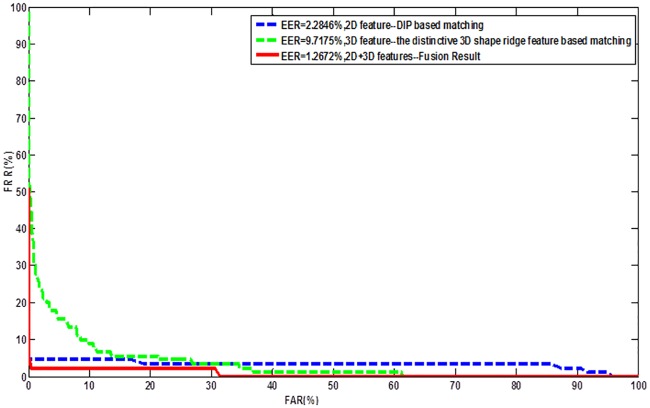
ROCs by matching 2D, 3D, and 2D+3D fingerprint features mentioned in this paper.

Fig 13 shows the ROCs achieved by matching DIP-based feature and the distinctive 3D shape ridge feature separately, as well as their combination. It is notable that the DIP-based feature clearly outperforms curve-skeleton in terms of accuracy. However, the best result is achieved when combining these two features where an EER of 1.3% is achieved. This experiment fully demonstrates that higher accuracy can be achieved if adding the distinctive 3D shape ridge feature. Thus, we concluded that this feature, at least, can be taken as an additional feature to current fingerprint feature set for user authentication.

### Comparisons with the art-of-the-state 3D fingerprint matching performance

Due to the lack of 3D fingerprint database, few studies reported the matching performance using 3D fingerprint images. Currently, the art-of-the-state 3D fingerprint recognition results were given in [13] and [14]. It is noted that different features, as well as different feature extraction and matching methods were adopted in their papers. What’s more, their results were obtained based on their own established database. In this paper, we summarized and compared the matching results using different features extracted from our own established 3D fingerprint database. The fusion results of these additional features with DIP based feature extracted from 2D fingerprint images were also given. Table 1 shows the EERs obtained by matching features mentioned in [13], [14] and this paper.

**Table 1:** Comparison of matching performance using the additional features extracted from 3D fingerprint images and fusion results with DIP feature extracted from 2D images.

Experiments	Equal Error Rate
curve-skeleton mentioned in [23]	13.87%
3D Curvature mentioned in [24]	11.95%
3D finger point cloud data using in this paper	16.85%
distinctive 3D shape ridge feature using in this paper	**9.72%**
DIP based feature+ curve-skeleton mentioned in [23]	1.88%
DIP based feature+ 3D Curvature mentioned in [24]	1.65%
DIP based feature+ 3D finger point cloud data using in this paper	2.96%
DIP based feature+ distinctive 3D shape ridge feature using in this paper	**1.27%**

From the results, it can be seen that the distinctiveness of all these features are poor. They are not suitable to be used alone to identify one’s identity. However, the accuracy is greatly improved when they are combined with 2D fingerprint features. Thus, there is no doubt that improved recognition accuracy can be achieved when 2D fingerprint features are combined with these additional features extracted from 3D fingerprint images. From Table 1, it also can be seen that the result of matching distinctive 3D shape ridge feature outperforms other features used in [13, 14], which further demonstrates the usefulness of the proposed distinctive 3D shape ridge feature.

## Conclusions

This paper had investigated the possible applications of 3D fingerprints. Thanks to the availability of 3D fingerprint images, more features can be extracted. The 3D finger shape features and the newly defined distinctive 3D shape ridge feature were then studied and extracted, the applications of these features were finally discussed as well. Results showed that coarse alignment could be easily achieved when 3D finger point cloud data were available. EER of ~8.3% can be obtained when the distinctive 3D shape ridge feature was used for personal authentication. Also, it was discovered to be helpful to remove false core point. Furthermore, promising EER was realized by combining this feature with 2D features for fingerprint recognition which indicated the prospect of 3D fingerprint recognition. In the paper, just simple feature extraction and matching methods were used. We believe that higher accuracy can be achieved if better feature extraction and matching methods are adopted. Thus, discovering more effective 3D feature extraction and matching methods (e.g. the 3D Gabor features, SSLBP features, improved SVM classifiers, sparse representation scheme, deep learning scheme etc. introduced in [30–40]) are our further work. Meanwhile, our future work includes exploring the relationship between different levels of fingerprint features and proposing more powerful fusion strategy. Information security is of great importance to current electronic age. 3D fingerprints provides more complicated features and increases dimensions of 2D fingerprint features which will be more suitable to embedded into the schemes of preventing attacks to current devices (e.g. 3D printer, multi-cloud-server) [41–46]. These will be our future direction to extend 3D fingerprints applications.

## Supporting information

S1 Database Part of the database used in the paper. (ZIP)

## References

[1] Maltoni D., Maio D., Jain A., and Prabhakar S.. Handbook of fingerprint recognition: New York, Springer Press; 2009.

[2] Parziale G, Diaz-Santana E, and Hauke R. The surround Imager™: a multi-camera touchless device to acquire 3d rolled-equivalent fingerprints. International Conference on Advances in Biometrics. Springer-Verlag, 2006: 244–250.

[3] A. Fatehpuria, D. Lau, and L. Hassebrook. Acquiring a 2-D rolled equivalent fingerprint image from a non-contact 3-D finger. in SPIE Defense and Security Symp. Biometric Technology for Human Identification III, Orlando, FL. 2006; 6202: 62020C-1–62020C-8.

[4] Xie W., Song Z., and Zhang X.. A Novel Photometric Method for Real-Time 3D Reconstruction of Fingerprint. Lecture Notes in Computer Science. 2010; 6454: 31–40.

[5] Wang Y., Lau D. L., and Hassebrook L. G.. Fit-sphere unwrapping and performance analysis of 3D fingerprints. Applied Optics. 2010; 49: 592–600. 10.1364/AO.49.000592 20119006

[6] Wang Y., Hassebrook L., and Lau D.. Data acquisition and processing of 3-D Fingerprints. IEEE Transactions on Information Forensics and Security. 2010; 5: 750–760.

[7] Troy M., Hassebrook L., Yalla V., and Daley R.. Non-contact 3D fingerprint scanner using Structured-light illumination. In SPIE MOEMS-MEMS. 2011: 79320C-1–79320C-13.

[8] TBS, 2013. http://www.tbs-biometrics.com.

[9] FlashScan, 2013. http://www.FlashScan3D.com.

[10] A. Kumar, and C. Kwong. Towards contactless, low-cost, and accurate 3D fingerprint identification. Proc. CVPR 2013, Portland, Oregon, 2013: 4321–4326. 10.1109/TPAMI.2014.2339818 26353269

[11] Pang X., Song Z., and Xie W.. Extraction of valley-ridge lines from the point cloud-based 3D fingerprint model. IEEE Computer Graphics and Applications. 2013; 33: 73–81. 10.1109/MCG.2012.128 24808061

[12] Huang S., Zhang Z., Zhao Y., Dai J., Chen C., and Xu Y., et al 3D fingerprint imaging system based on full-field fringe projection profilometry. Optics and Lasers in Engineering. 2014; 52: 123–130.

[13] Liu F., Zhang D., and Shen L.. Study on novel Curvature Features for 3D fingerprint recognition. Neurocomputing, 2015; 168: 599–608.

[14] Kumar Ajay, and Kwong Cyril. Towards contactless, low-cost and accurate 3D fingerprint identification. IEEE Trans. Pattern Analysis & Machine Intelligence. 2015; 37: 681–696. 10.1109/TPAMI.2014.2339818 26353269

[15] Zhang D., Kanhangad V., Luo N., and Kumar A.. Robust palmprint verification using 2D and 3D features. Pattern Recognition. 2010; 43: 358–368.

[16] Srinivassan V., and Liu H.. Automated phase measuring profilometry of 3D diffuse object. Applied Optics. 1984; 23: 3105–3108. 18213131 10.1364/ao.23.003105

[17] Liu F., and Zhang D.. 3D fingerprint reconstruction system using feature correspondences and finger shape model. Pattern Recognition. 2014; 47: 178–193.

[18] NetPreProc. Max-min graph normalization. http://artax.karlin.mff.cuni.cz/rhelp/library/NetPreProc/html/Max.Min.norm-methods.html.

[19] Besl P. J., and McKay N. D.. A method for registration of 3-D shapes. IEEE Trans. Pattern Anal. Mach. Intell. 1992; 14: 239–256.

[20] Zhang D., and Lu G.M.. 3D biometrics. 1st ed Springer New York Press; 2013 pp. 171–230.

[21] F. Liu. New generation of automated fingerprint recognition system, electronic, doctoral dissertations. The Hong Kong Polytechnic University, 2014. http://ira.lib.polyu.edu.hk/bitstream/10397/6878/2/b26961064_ir.pdf.

[22] G. Parziale, and A. Niel. A fingerprint matching using minutiae triangulation. on Proc. of International Conference on Biometric Authentication (ICBA), LNCS. 2004; 3072: 241–248.

[23] Liu L., Fingerprint orientation alignment and similarity measurement. The Imaging Science Journal. 2007; 55: 114–125.

[24] Lindoso A., Entrena L., Liu-Jimenez J., and Millan E.. Correlation-based fingerprint matching with orientation field alignment. Advances in Biometrics. 2007; 4642: 713–721.

[25] W. Li, N. Bhattacharjee, and B. Srinivasan. A method for fingerprint alignment and matching. Proceedings of the 10th International Conference on Advances in Mobile Computing & Multimedia, ACM. 2012: 297–301.

[26] Yager N., and Amin A.. Fingerprint alignment using a two stage optimization. Pattern Recognition Letters. 2006; 27: 317–324.

[27] Zhao Q., Zhang D., Zhang L., and Luo N.. High resolution partial fingerprint alignment using pore–valley descriptors. Pattern Recognition. 2010; 43: 1050–1061.

[28] Liu F., Zhang D., and Guo Z.. Distal Interphalangeal Crease based User Authentication System. IEEE T. Information Forensics and Security. 2013; 8: 1446–1455.

[29] A.W. Kong, and D. Zhang. Competitive coding scheme for palmprint verificatio. in Proc. 17th Int. Conf. Pattern Recognition. 2004; 1: 520–523.

[30] Wong W., Lai Z., Xu Y., and Wen J.. Joint tensor feature analysis for visual object recognition. IEEE Transactions on Cybernetics. 2015; 45: 2425–2436. 10.1109/TCYB.2014.2374452 26470058

[31] Zhu Z., Jia S., He S., Sun Y., Ji Z., and Shen L.. Three-dimensional gabor feature extraction for hyperspectral imagery classification using a memetic framework. Information Sciences. 2015; 298: 274–287.

[32] Shi X., Guo Z., and Lai Z.. Face recognition by sparse discriminant analysis via joint L2,1-norm minimizatio. Pattern Recognition. 2014; 47: 2447–2453.

[33] Shen L., Bai L., and Ji Z.. FPCODE: An efficient approach for multi-modal biometrics. International Journal of Pattern Recognition and Artificial Intelligence. 2015; 25: 273–286.

[34] Lu X., Yuan Y., and Zheng X. Joint dictionary learning for multispectral change detection. IEEE Transactions on Cybernetics. 2016; 46: 1–14. 10.1109/TCYB.2016.2531179 26955060

[35] Yuan Y., Mou L., and Lu X.. Scene recognition by manifold regularized deep learning architecture. IEEE Transactions on Neural Networks and Learning Systems. 2015; 26: 2222–2233. 10.1109/TNNLS.2014.2359471 25622326

[36] Guo Z., Wang X., Zhou J., and You J.. Robust texture image representation by scale selective local binary patterns (SSLBP). IEEE Transactions on Image Processing. 2016; 25: 687–699. 10.1109/TIP.2015.2507408 26685235

[37] Shi X., Guo Z., Lai Z., Yang Y., Bao Z. and Zhang D.. A framework of joint graph embedding and sparse regression for dimensionality reduction. IEEE Transactions on Image Processing, 2015; 24: 1341–1355. 10.1109/TIP.2015.2405474 25706635

[38] Gu B., and Sheng Victor S., A robust regularization path algorithm for ν-Support vector classification. IEEE Transactions on Neural Networks and Learning Systems. 2016; 1: 1–8. 10.1109/TNNLS.2016.2527796 26929067

[39] Gu B., Sheng Victor S., Tay Keng Yeow, Romano Walter, and Shuo Li. Incremental support vector learning for ordinal regression. IEEE Transactions on Neural Networks and Learning Systems. 2015; 26: 1403–1416. 10.1109/TNNLS.2014.2342533 25134094

[40] Gu Bin, Sheng Victor S., Wang Zhijie, Ho Derek, Osman Said, and Li Shuo. Incremental learning for ν-Support vector regression. Neural Networks. 2015; 67: 140–150. 10.1016/j.neunet.2015.03.013 25933108

[41] Do Quang, Martini Ben, and Choo Kim-Kwang Raymond. A data exfiltration and remote exploitation attack on consumer 3D printers. IEEE Trans. Information Forensics and Security. 2016; 11: 2174–2186.

[42] Kumari S, Xiong L, Wu F, Das A K, Choo K-K R, and Shen J. Design of a provably secure biometrics-based multi-cloud-server authentication scheme. Future Generation Computer Systems. 2017; 6: 320–330.

[43] Peng J, Choo K-K R, and Ashman H. User profiling in intrusion detection: A review. Journal of Network and Computer Applications. 2016; 72: 14–27.

[44] Yuan Chengsheng, Sun Xingming, and Rui LV. Fingerprint liveness detection based on multi-scale LPQ and PCA. China Communications. 2016; 13: 60–65.

[45] Zhou Zhili, Wang Yunlong, Wu Q.M. Jonathan, Yang Ching-Nung, and Sun Xingming. Effective and ffficient global context verification for image copy detectio. IEEE Transactions on Information Forensics and Security. 2017; 12: 48–63.

[46] Fu Zhangjie, Wu Xinle, Guan Chaowen, Sun Xingming, and Ren Kui. Toward efficient multi-keyword fuzzy search over encrypted outsourced data with accuracy improvement. IEEE Transactions on Information Forensics and Security. 2016; 11: 2706–2716.

