# Supplementary figures and images for: Case study of 3D fingerprints applications
Source: PLoS One. 2017 Apr 11;12(4):e0175261. doi: 10.1371/journal.pone.0175261 (PMC5388323; doi:10.1371/journal.pone.0175261)

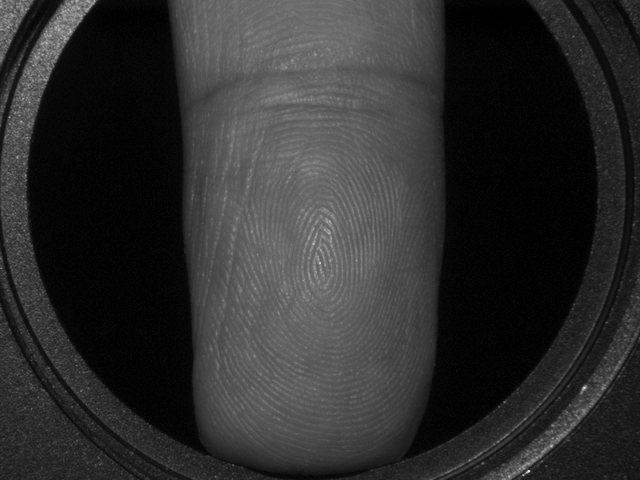

Supplement: S1 Database — (ZIP) [file pone.0175261.s001.zip › S1_Database/S1_Database_Session1-2D texture fingreprint images/bz_b_1.bmp]

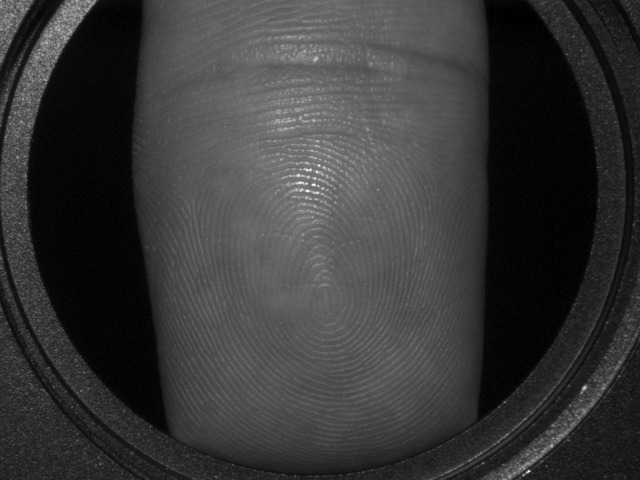

Supplement: S1 Database — (ZIP) [file pone.0175261.s001.zip › S1_Database/S1_Database_Session1-2D texture fingreprint images/bz_l1_b_1.bmp]

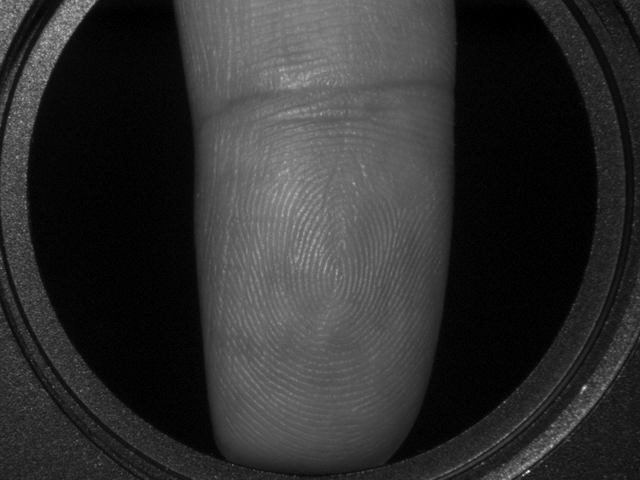

Supplement: S1 Database — (ZIP) [file pone.0175261.s001.zip › S1_Database/S1_Database_Session1-2D texture fingreprint images/bz_l2_b_1.bmp]

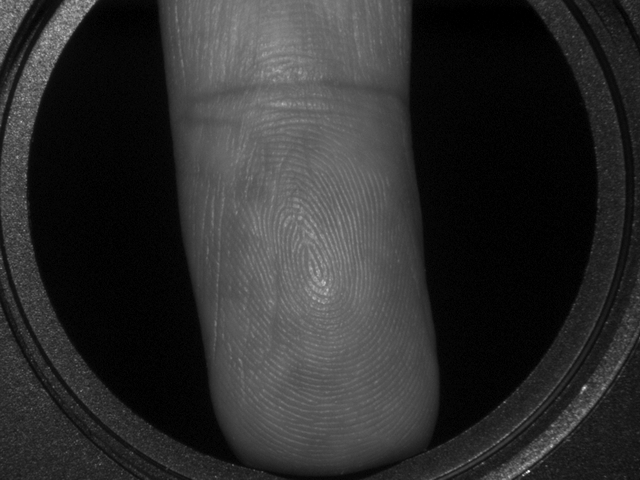

Supplement: S1 Database — (ZIP) [file pone.0175261.s001.zip › S1_Database/S1_Database_Session1-2D texture fingreprint images/bz_l4_b_1.bmp]

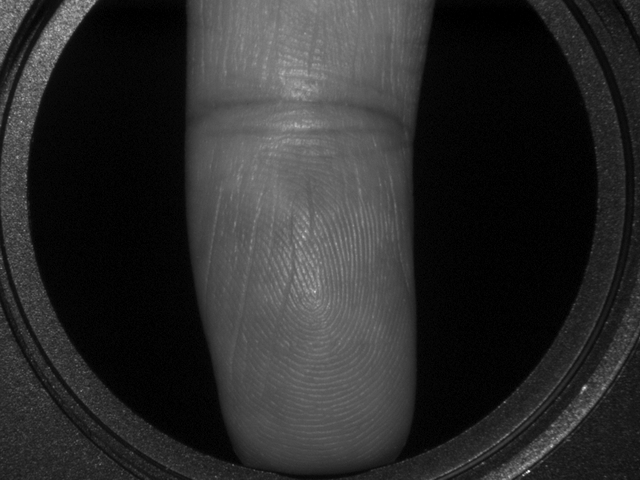

Supplement: S1 Database — (ZIP) [file pone.0175261.s001.zip › S1_Database/S1_Database_Session1-2D texture fingreprint images/bz_l5_b_1.bmp]

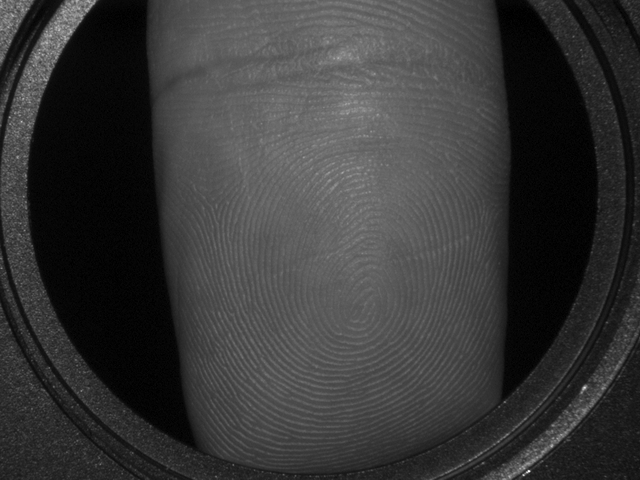

Supplement: S1 Database — (ZIP) [file pone.0175261.s001.zip › S1_Database/S1_Database_Session1-2D texture fingreprint images/bz_r1_b_1.bmp]

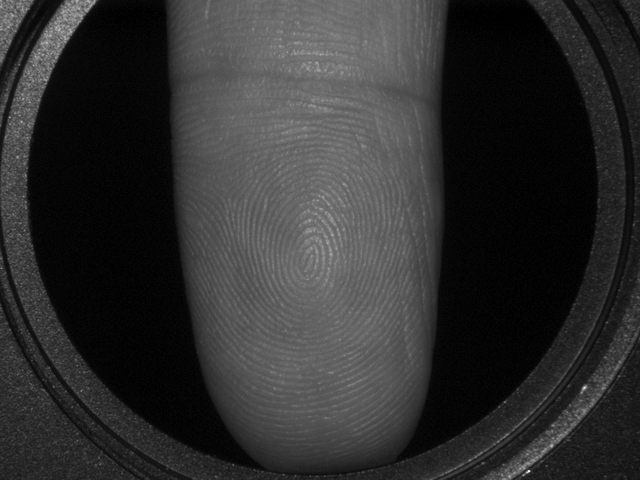

Supplement: S1 Database — (ZIP) [file pone.0175261.s001.zip › S1_Database/S1_Database_Session1-2D texture fingreprint images/bz_r2_b_1.bmp]

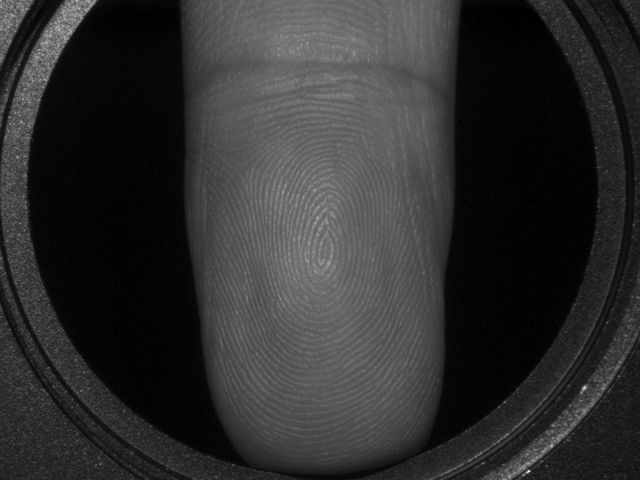

Supplement: S1 Database — (ZIP) [file pone.0175261.s001.zip › S1_Database/S1_Database_Session1-2D texture fingreprint images/bz_r3_b_1.bmp]

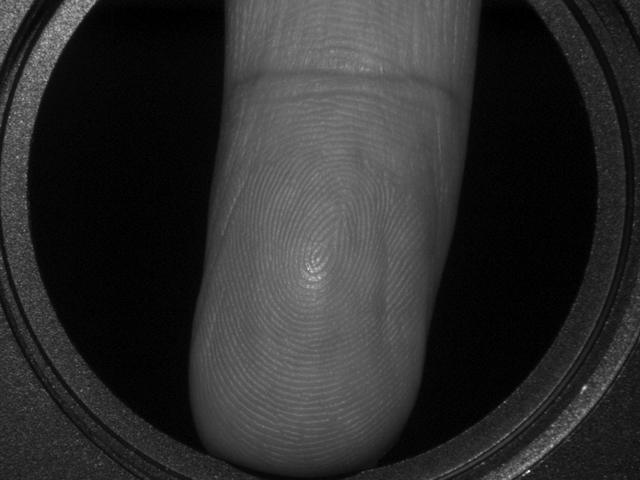

Supplement: S1 Database — (ZIP) [file pone.0175261.s001.zip › S1_Database/S1_Database_Session1-2D texture fingreprint images/bz_r4_b_1.bmp]

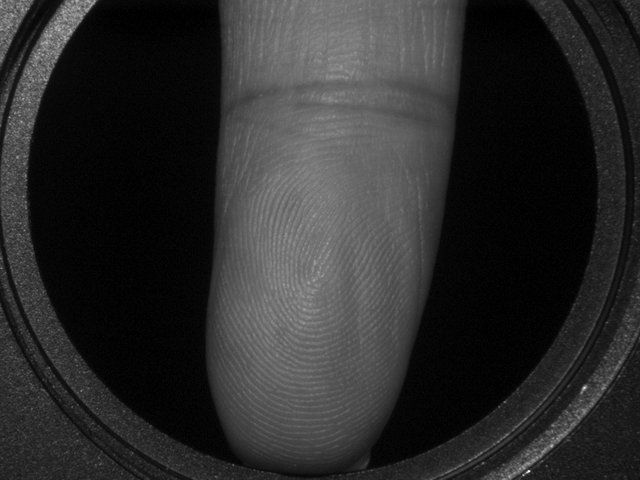

Supplement: S1 Database — (ZIP) [file pone.0175261.s001.zip › S1_Database/S1_Database_Session1-2D texture fingreprint images/bz_r5_b_1.bmp]

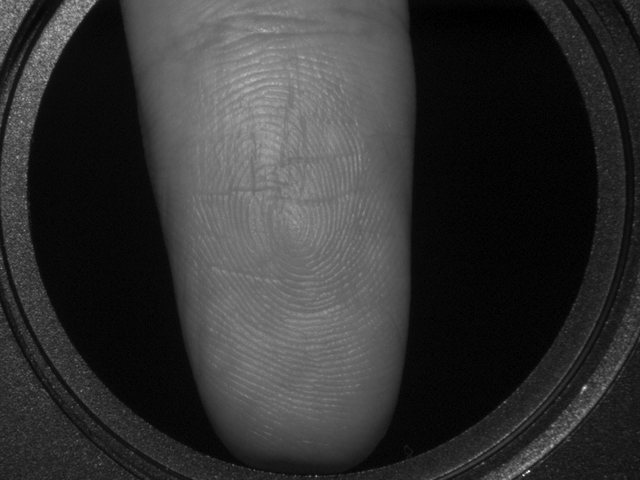

Supplement: S1 Database — (ZIP) [file pone.0175261.s001.zip › S1_Database/S1_Database_Session1-2D texture fingreprint images/cjr_l1_g_1.bmp]

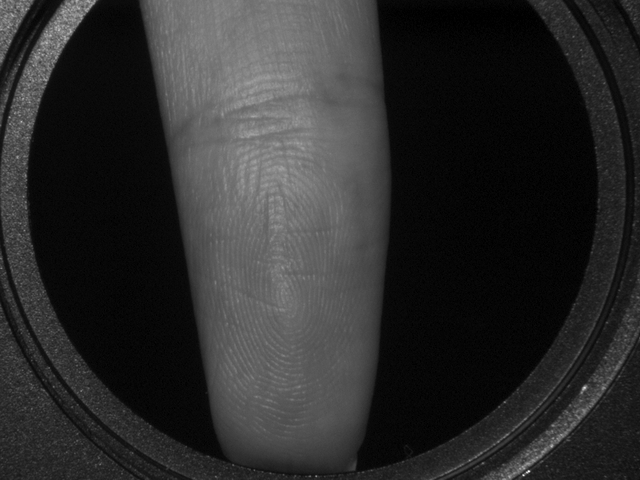

Supplement: S1 Database — (ZIP) [file pone.0175261.s001.zip › S1_Database/S1_Database_Session1-2D texture fingreprint images/cjr_l2_g_1.bmp]

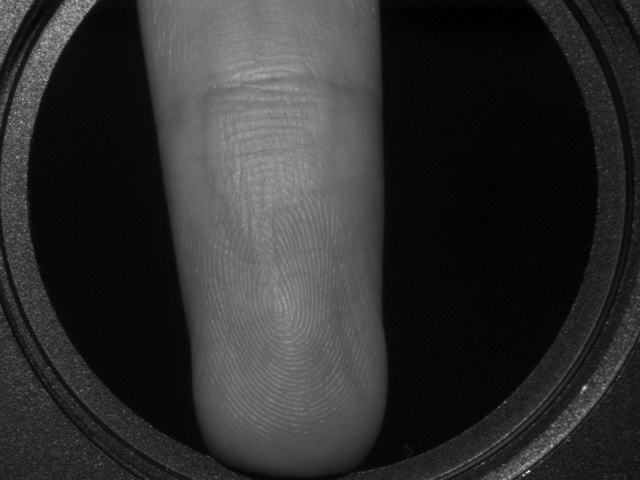

Supplement: S1 Database — (ZIP) [file pone.0175261.s001.zip › S1_Database/S1_Database_Session1-2D texture fingreprint images/cjr_l3_g_1.bmp]

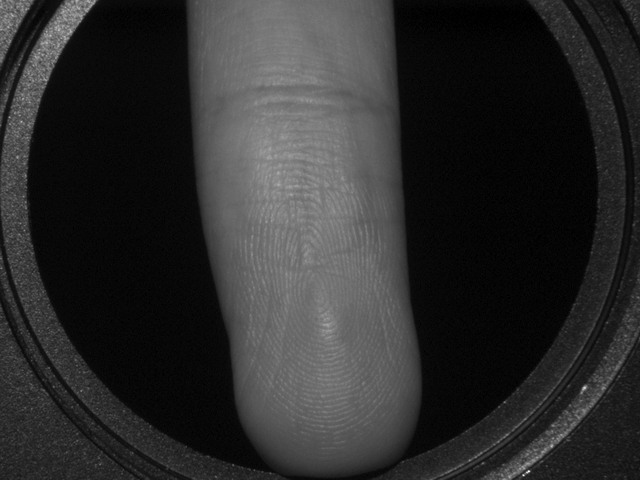

Supplement: S1 Database — (ZIP) [file pone.0175261.s001.zip › S1_Database/S1_Database_Session1-2D texture fingreprint images/cjr_l4_g_1.bmp]

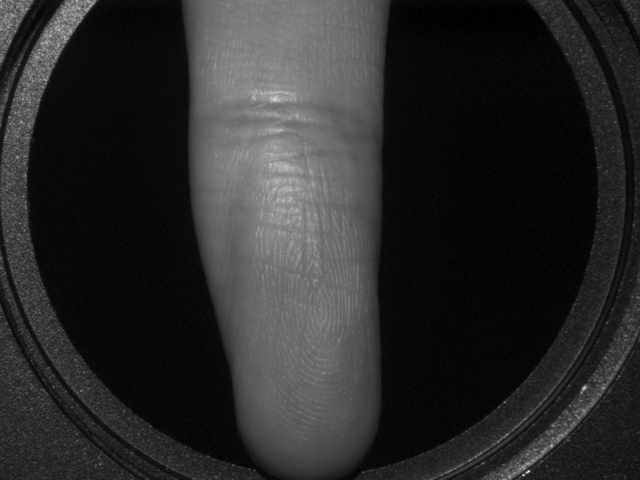

Supplement: S1 Database — (ZIP) [file pone.0175261.s001.zip › S1_Database/S1_Database_Session1-2D texture fingreprint images/cjr_l5_g_1.bmp]

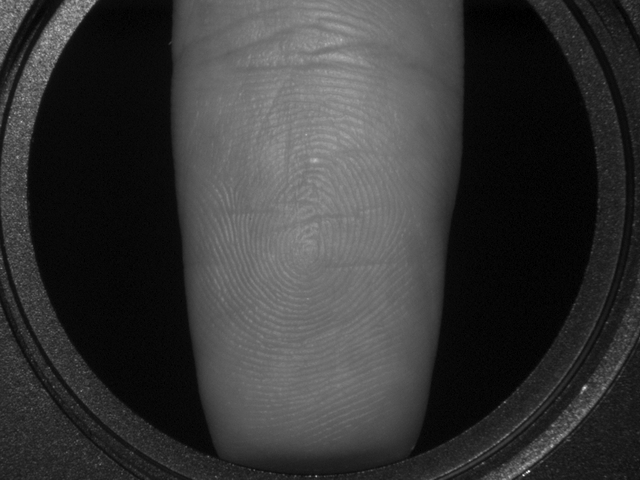

Supplement: S1 Database — (ZIP) [file pone.0175261.s001.zip › S1_Database/S1_Database_Session1-2D texture fingreprint images/cjr_r1_g_1.bmp]

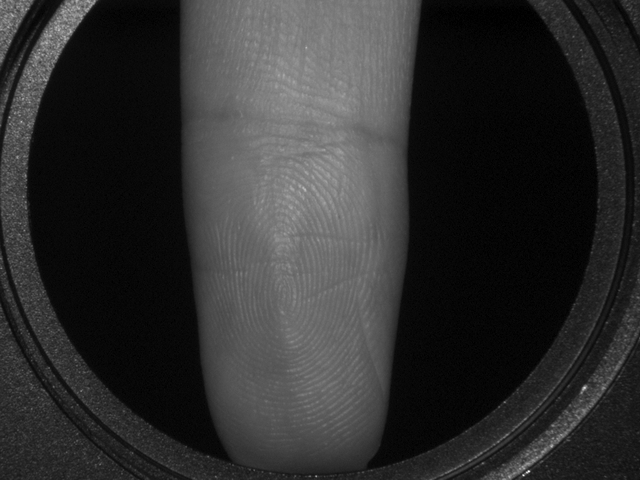

Supplement: S1 Database — (ZIP) [file pone.0175261.s001.zip › S1_Database/S1_Database_Session1-2D texture fingreprint images/cjr_r2_g_1.bmp]

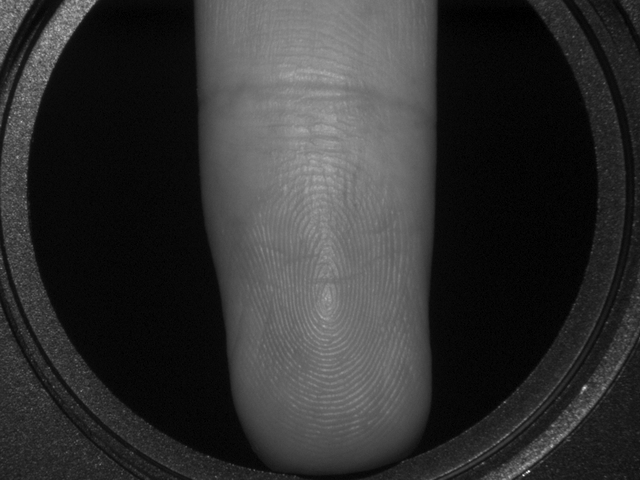

Supplement: S1 Database — (ZIP) [file pone.0175261.s001.zip › S1_Database/S1_Database_Session1-2D texture fingreprint images/cjr_r3_g_1.bmp]

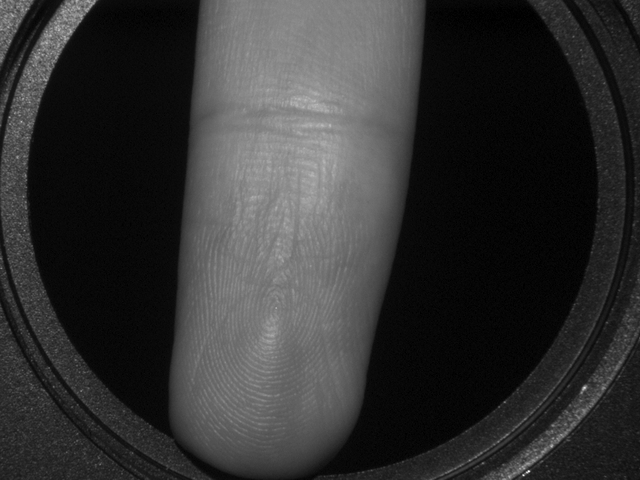

Supplement: S1 Database — (ZIP) [file pone.0175261.s001.zip › S1_Database/S1_Database_Session1-2D texture fingreprint images/cjr_r4_g_1.bmp]

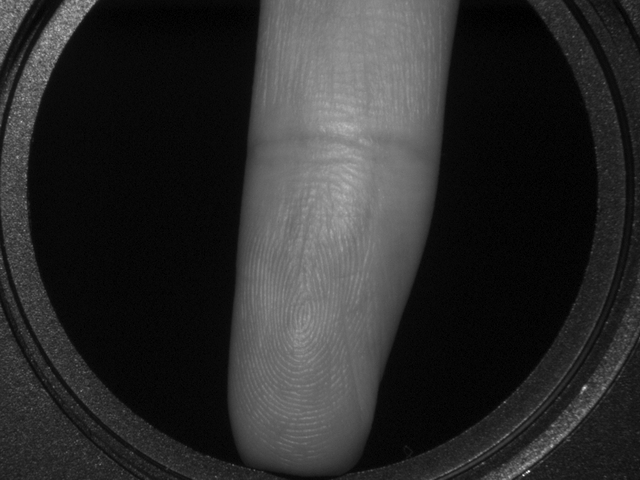

Supplement: S1 Database — (ZIP) [file pone.0175261.s001.zip › S1_Database/S1_Database_Session1-2D texture fingreprint images/cjr_r5_g_1.bmp]

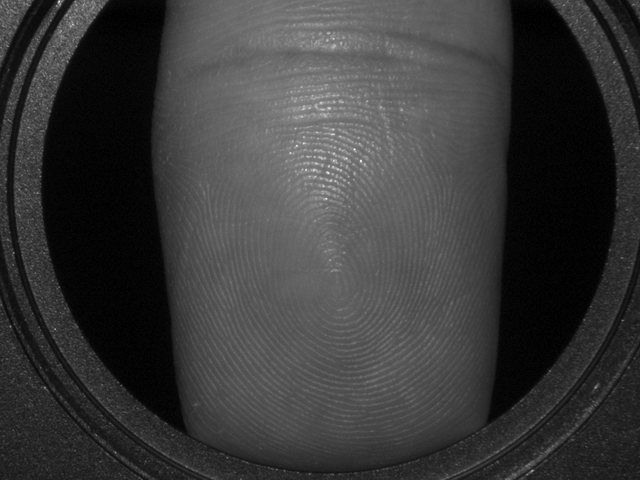

Supplement: S1 Database — (ZIP) [file pone.0175261.s001.zip › S1_Database/S1_Database_Session2-2D texture fingreprint images/bz_l1_b_2.bmp]

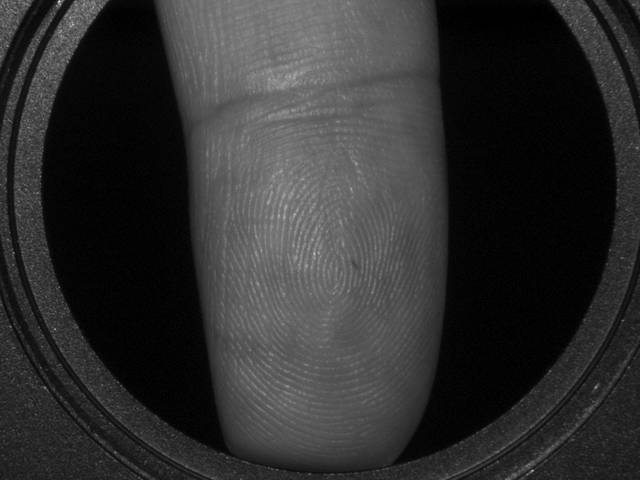

Supplement: S1 Database — (ZIP) [file pone.0175261.s001.zip › S1_Database/S1_Database_Session2-2D texture fingreprint images/bz_l2_b_2.bmp]

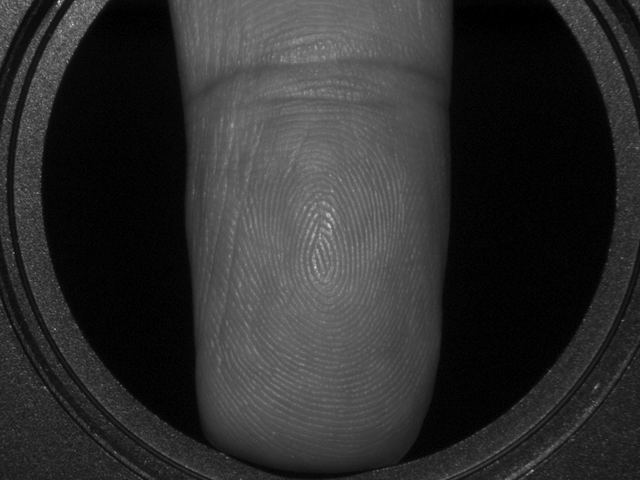

Supplement: S1 Database — (ZIP) [file pone.0175261.s001.zip › S1_Database/S1_Database_Session2-2D texture fingreprint images/bz_l3_b_2.bmp]

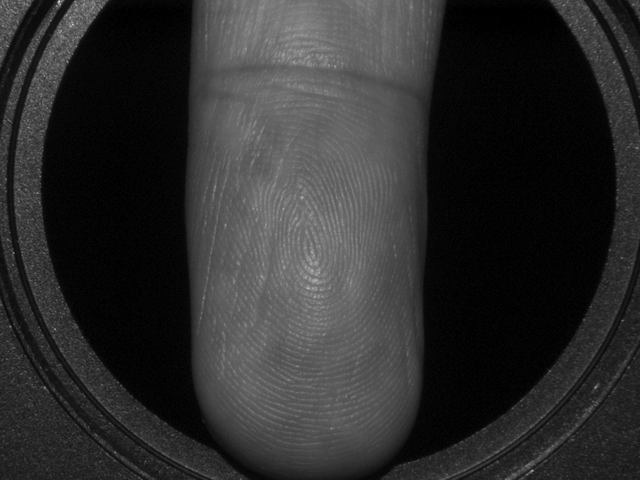

Supplement: S1 Database — (ZIP) [file pone.0175261.s001.zip › S1_Database/S1_Database_Session2-2D texture fingreprint images/bz_l4_b_2.bmp]

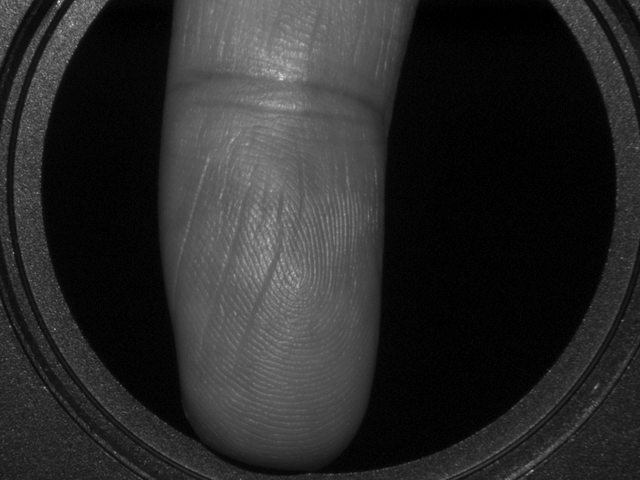

Supplement: S1 Database — (ZIP) [file pone.0175261.s001.zip › S1_Database/S1_Database_Session2-2D texture fingreprint images/bz_l5_b_2.bmp]

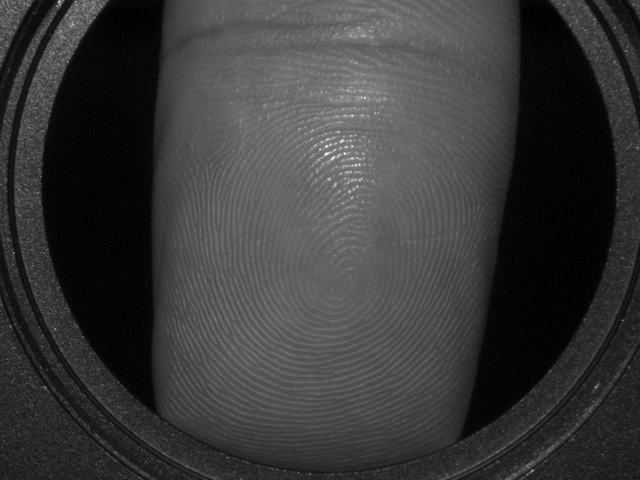

Supplement: S1 Database — (ZIP) [file pone.0175261.s001.zip › S1_Database/S1_Database_Session2-2D texture fingreprint images/bz_r1_b_2.bmp]

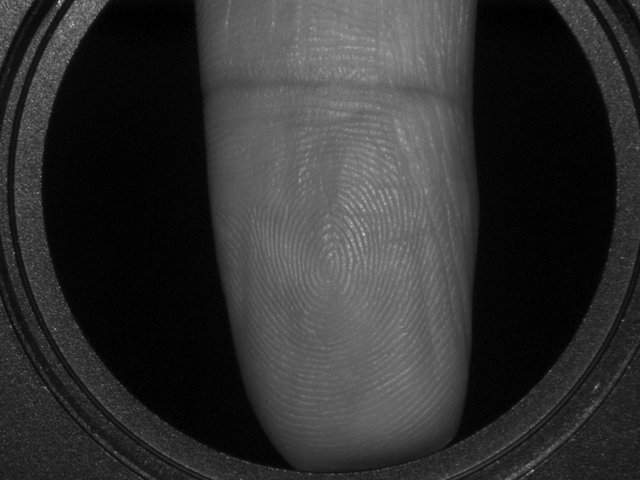

Supplement: S1 Database — (ZIP) [file pone.0175261.s001.zip › S1_Database/S1_Database_Session2-2D texture fingreprint images/bz_r2_b_2.bmp]

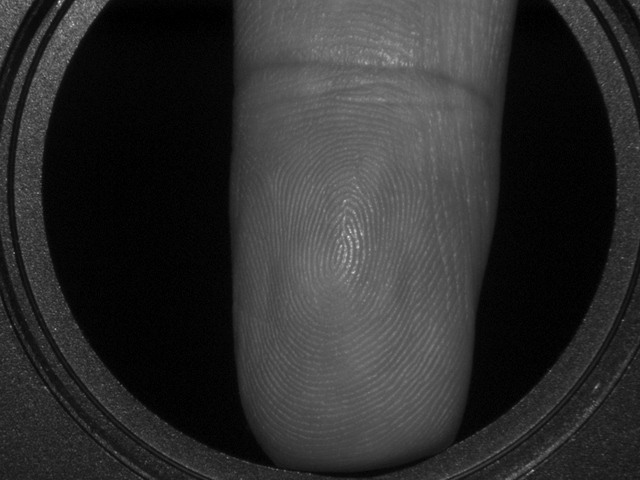

Supplement: S1 Database — (ZIP) [file pone.0175261.s001.zip › S1_Database/S1_Database_Session2-2D texture fingreprint images/bz_r3_b_2.bmp]

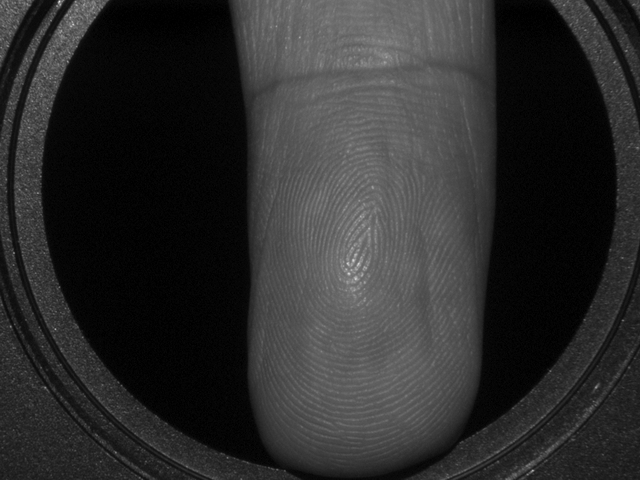

Supplement: S1 Database — (ZIP) [file pone.0175261.s001.zip › S1_Database/S1_Database_Session2-2D texture fingreprint images/bz_r4_b_2.bmp]

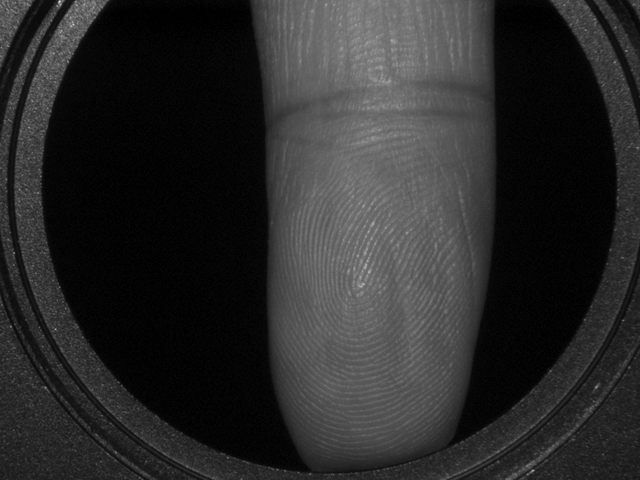

Supplement: S1 Database — (ZIP) [file pone.0175261.s001.zip › S1_Database/S1_Database_Session2-2D texture fingreprint images/bz_r5_b_2.bmp]

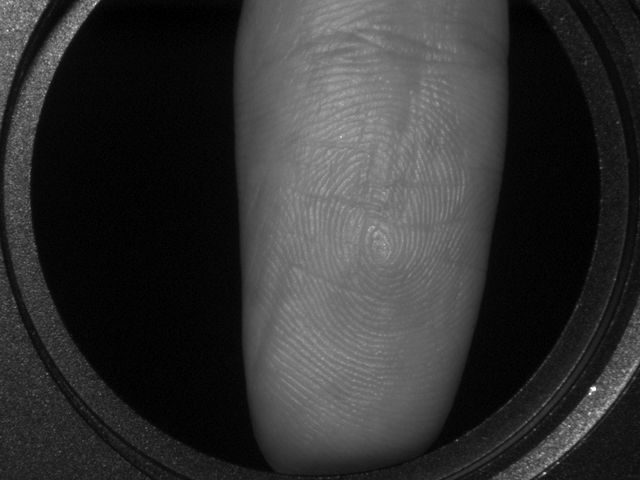

Supplement: S1 Database — (ZIP) [file pone.0175261.s001.zip › S1_Database/S1_Database_Session2-2D texture fingreprint images/cjr_l1_g_2.bmp]

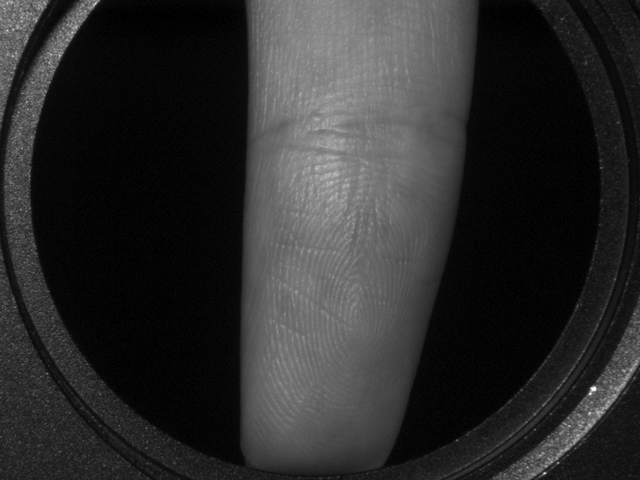

Supplement: S1 Database — (ZIP) [file pone.0175261.s001.zip › S1_Database/S1_Database_Session2-2D texture fingreprint images/cjr_l2_g_2.bmp]

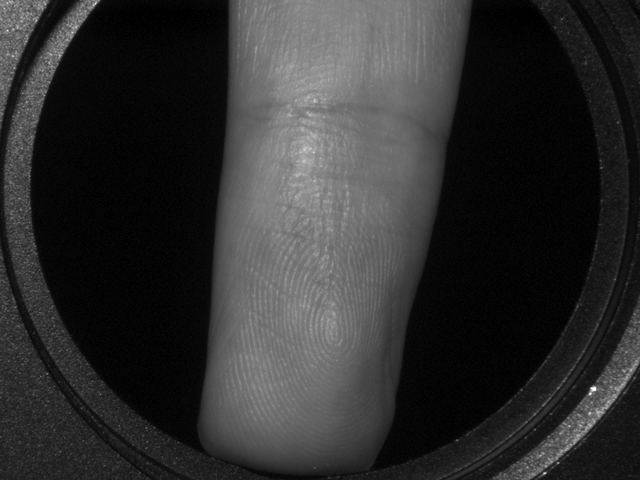

Supplement: S1 Database — (ZIP) [file pone.0175261.s001.zip › S1_Database/S1_Database_Session2-2D texture fingreprint images/cjr_l3_g_2.bmp]

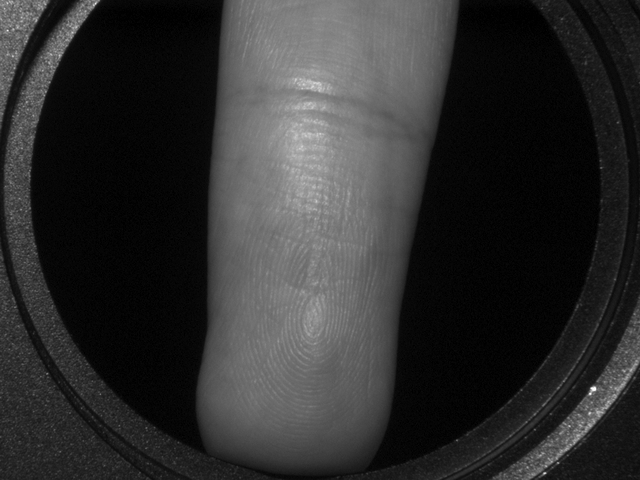

Supplement: S1 Database — (ZIP) [file pone.0175261.s001.zip › S1_Database/S1_Database_Session2-2D texture fingreprint images/cjr_l4_g_2.bmp]

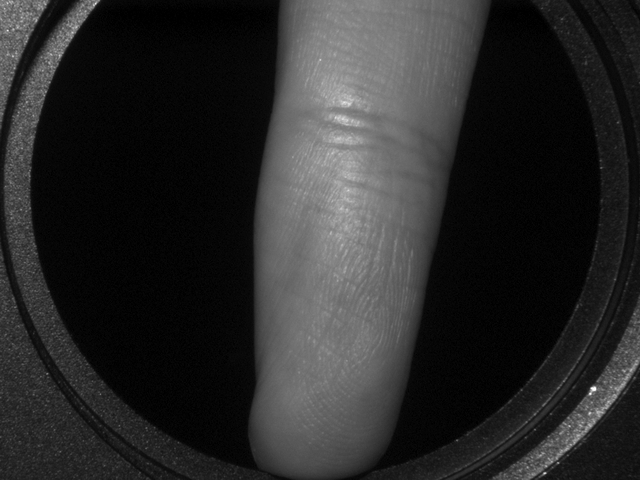

Supplement: S1 Database — (ZIP) [file pone.0175261.s001.zip › S1_Database/S1_Database_Session2-2D texture fingreprint images/cjr_l5_g_2.bmp]

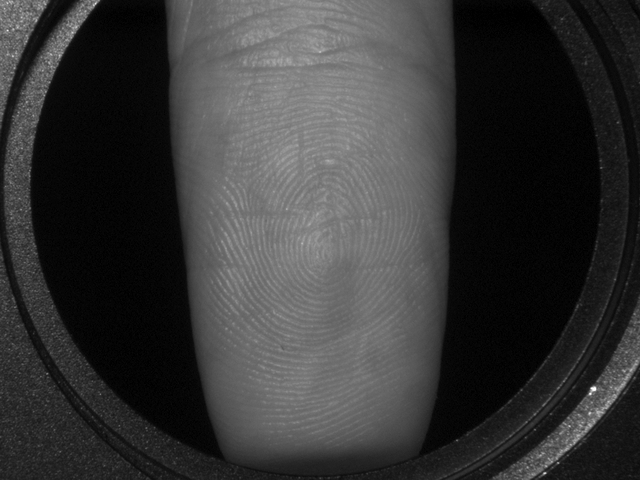

Supplement: S1 Database — (ZIP) [file pone.0175261.s001.zip › S1_Database/S1_Database_Session2-2D texture fingreprint images/cjr_r1_g_2.bmp]

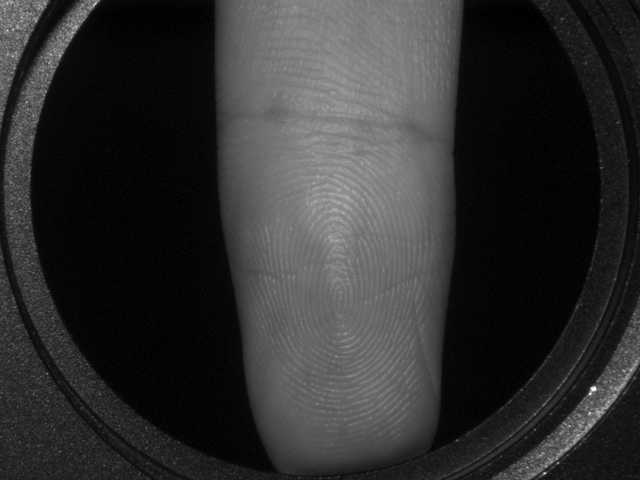

Supplement: S1 Database — (ZIP) [file pone.0175261.s001.zip › S1_Database/S1_Database_Session2-2D texture fingreprint images/cjr_r2_g_2.bmp]

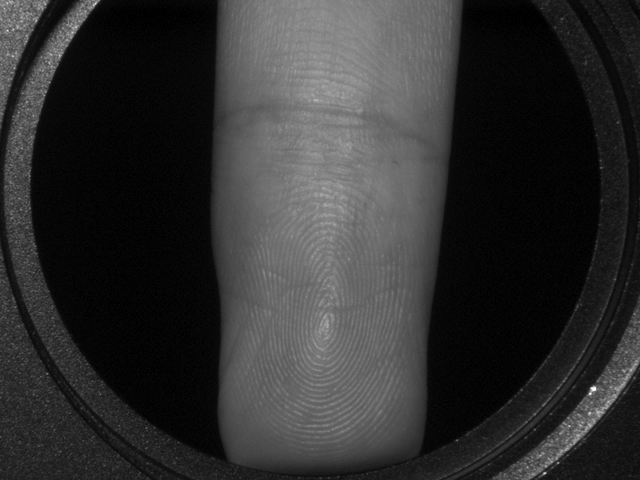

Supplement: S1 Database — (ZIP) [file pone.0175261.s001.zip › S1_Database/S1_Database_Session2-2D texture fingreprint images/cjr_r3_g_2.bmp]

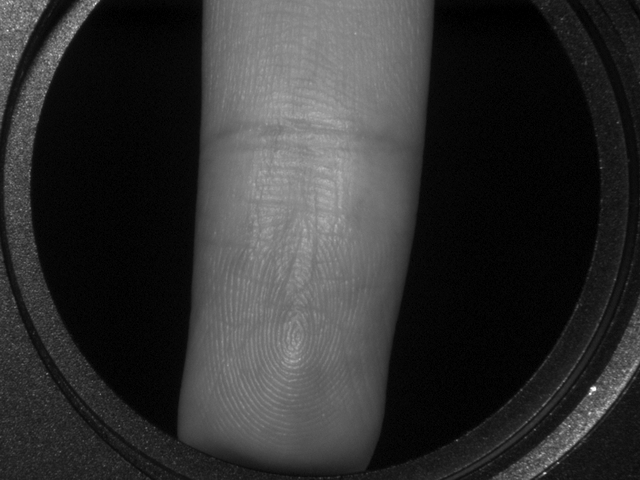

Supplement: S1 Database — (ZIP) [file pone.0175261.s001.zip › S1_Database/S1_Database_Session2-2D texture fingreprint images/cjr_r4_g_2.bmp]

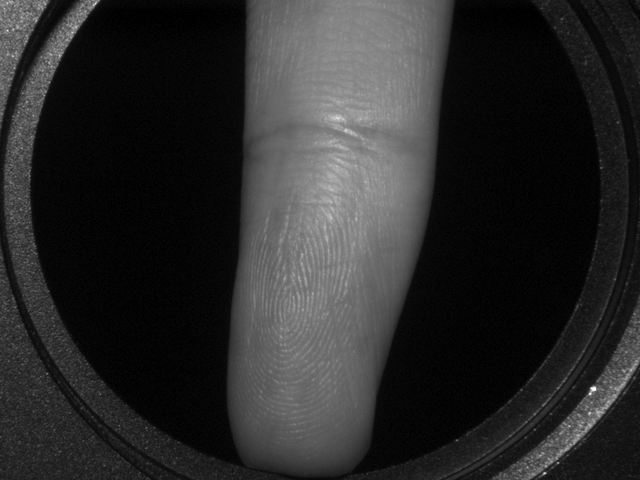

Supplement: S1 Database — (ZIP) [file pone.0175261.s001.zip › S1_Database/S1_Database_Session2-2D texture fingreprint images/cjr_r5_g_2.bmp]
